# Mechanisms of Antibiotic Resistance and Novel Therapeutic Approaches for *Mycobacterium tuberculosis*: A Narrative Review With a Focus on Tuberculosis Mutations in Iran

**DOI:** 10.1002/hsr2.71313

**Published:** 2025-10-08

**Authors:** Ali Bayat Bodaghi, Aref Shariati, Jebreil Shamseddin, Amir Bayat Bodaghi, Mina Rezaei, Abbas Farahani

**Affiliations:** ^1^ Student Research Committee Khomein University of Medical Sciences Khomein Iran; ^2^ Infectious Diseases Research Center (IDRC) Arak University of Medical Sciences Arak Iran; ^3^ Infectious and Tropical Diseases Research Center, Hormozgan Health Institute Hormozgan University of Medical Sciences Bandar Abbas Iran; ^4^ Student Research Committee, School of Medicine Zanjan University of Medical Sciences Zanjan Iran; ^5^ School of Environment, College of Engineering University of Tehran Tehran Iran; ^6^ Molecular Medicine Research Center Khomein University of Medical Sciences Khomein Iran; ^7^ Department of Basic Sciences Khomein University of Medical Sciences Khomein Iran

**Keywords:** antibiotic resistance, *Mycobacterium tuberculosis*, nanoparticles, treatment

## Abstract

**Background and Aims:**

Tuberculosis, caused by *Mycobacterium tuberculosis* (*M. tuberculosis*), is a highly contagious disease and one of the leading causes of mortality globally. The emergence and dissemination of multidrug‐resistant (MDR) strains of *M. tuberculosis* have become a major concern, especially in developing countries. Antibiotic resistance in bacteria constitutes a substantial threat to human health, both now and in the future. The current treatment regimens for tuberculosis are challenging and demanding. Consequently, understanding the antibiotic resistance mechanisms of *M. tuberculosis* is of paramount importance for global health. This review provides a comprehensive overview of the present state of *M. tuberculosis* antibiotic resistance by elucidating the underlying mechanisms and presenting up‐to‐date information and findings in the field. It also highlights deficiencies and challenges in existing studies, with particular emphasis on tuberculosis mutations in the Iranian studies. Furthermore, the study investigate into contemporary global treatment modalities and illuminates new therapeutic avenues for tuberculosis management.

**Method:**

This study conducts a review of the literature on *M. tuberculosis* and its antibiotic resistance, using databases such as Scopus, Embase, MEDLINE/PubMed, Web of Science, and Google Scholar.

**Results:**

The current status of antibiotic resistance in *M. tuberculosis* represents a serious threat to global health. MDR and extensively drug‐resistant (XDR) strains of *M. tuberculosis* are becoming increasingly widespread.

**Conclusion:**

The increasing antibiotic resistance in *M. tuberculosis* constitutes a significant global health threat, with MDR and XDR strains becoming more prevalent. Effective responses require the development of novel therapeutic drugs, ensuring patients complete their full drug treatment courses, implementing innovative strategies to prevent resistance, and accelerating the creation of effective compounds. Large‐scale studies on diverse populations are essential to develop comprehensive solutions and combat the spread of resistance in tuberculosis. Urgent and coordinated efforts are needed to enhance treatment efficacy and control the spread of drug‐resistant TB.

## 1 Introduction

*Tuberculosis*, caused by *Mycobacterium tuberculosis* (*M. tuberculosis*), and first identified by Robert Koch, remains a major contributor to global mortality. The disease is highly contagious and continues to be prevalent, particularly in developing countries [1, 2, 3]. *M. tuberculosis* is one of the most fatal human pathogens. In 2022, an estimated 28,000 individuals will acquire the infection daily, caused 4100 daily deaths [4, 5].

*Tuberculosis* is categorized into latent tuberculosis, characterized by asymptomatic and noncontagious infection, and active tuberculosis, characterized by symptoms such as fever, weight loss, hemoptysis, and persistent cough, along with contagiousness [3]. Antibiotics are substances that exert their bactericidal or bacteriostatic effects by targeting crucial biochemical mechanisms of microorganisms. Antibiotic resistance is defined as the ability of a pathogen to survive and proliferate within the host's body despite exposure to antibiotics, which under natural circumstances should have eradicated the pathogen [6]. Antibiotic resistance can arise from physiological, intrinsic, or acquired factors. Intrinsic resistance occurs due to factors such as the cell wall composition, efflux systems, drug‐deactivating enzymes in the cytoplasm, and low drug affinity. Acquired resistance, on the other hand, is typically the result of chromosomal mutations [7]. The rapid spread of antibiotic resistance in *M. tuberculosis* has heightened global concerns regarding tuberculosis [8]. In 2019, approximately 0.5 million cases of antibiotic‐resistant tuberculosis were documented, with nearly 78% of these cases demonstrating resistance to multiple antibiotics [1].

Tuberculosis treatment is notably longer than that for many other infectious diseases, typically extending from approximately 6 to 9 months. Therapeutic regimens involve a combination of multiple antituberculous agents. A central challenge in treating tuberculosis is the pathogen itself, *M. tuberculosis*, which can acquire mutations and develop antibiotic resistance within the host. [9, 10]. The current standard therapeutic regimen for active tuberculosis includes the administration of *isoniazid* (INH), *rifampicin* (RIF), *ethambutol* (EMB), and *pyrazinamide* (PZA) for the initial 2 months, followed by INH and RIF for the subsequent 4 months [3]. Failure to complete the full treatment course increases the risk of disease recurrence and the emergence of drug resistance, complicating management and requiring longer, more expensive, and potentially more toxic second‐line therapies [3, 11]. In cases of drug‐resistant tuberculosis (DR‐TB) strains, the treatment duration can extend to approximately 18 months or longer, necessitating the use of more costly second‐line drugs that exhibit less certain effectiveness and greater toxicity compared to the first‐line drugs [3]. The treatment of tuberculosis is further complicated by the unique ability of the bacterium *M. tuberculosis* to survive within the human body and inside macrophages. As previously mentioned, drug resistance poses a significant problem in tuberculosis treatment and can occur via either hereditary or acquired mechanisms [12]. MDR strains (multi‐drug resistant) of *M. tuberculosis* are defined as those that are resistant to at least the first‐line drugs, including INH and RIF [11, 13]. On average, around 5% of newly diagnosed tuberculosis cases fall into this category [13]. Another type of drug‐resistant strain, known as extensively drug‐resistant (XDR), is resistant to INH, RIF, at least one of the second‐line injectable drugs such as amikacin, kanamycin, or capromycin, as well as any fluoroquinolone [13]. It is estimated that around 9% of MDR strains progress to become XDR strains [14].

Bacterial antibiotic resistance represents a critical threat to human health today. The rise and dissemination of multidrug‐resistant (MDR) strains pose ongoing challenges to the treatment of bacterial infections [15]. While tuberculosis is now a curable disease, its treatment remains intricate and demanding [16]. Given the escalating prevalence of antibiotic‐resistant strains, the antibiotic resistance of *M. tuberculosis* assumes paramount importance in global health [7].

This review aims to offer a comprehensive insight into the current landscape of *M. tuberculosis* antibiotic resistance by delving into the underlying mechanisms and updating the knowledge and discoveries in this domain. It also seeks to address prevailing gaps and issues, particularly emphasis on tuberculosis mutations in Iranian studies, and suggest potential remedies. Furthermore, the study investigate into contemporary global treatment modalities and illuminates new therapeutic avenues for tuberculosis management.

## 2 Resistance Mechanisms

The emergence and progression of resistant strains of *M. tuberculosis* are principally driven by the selection of genetic mutations. Under selective pressure, the bacterial population expands variants that confer survival advantages, leading to the fixation of antibiotic resistance as a dominant trait. Both genetic and phenotypic resistance are commonly observed types. Additionally, chromosomal gene mutations result in genetic changes that contribute to the development of drug resistance in the bacteria. The primary mechanism of *M. tuberculosis* could be classified into intrinsic and acquired resistance.

Intrinsic resistance:

*Mycobacterium* species exhibit reduced permeability to therapeutic agents due to their complex cell wall structure, which includes mycolic acids. Prolonged exposure to subinhibitory drug concentrations can induce activation and overexpression of transporter proteins, potentially leading to irreversible phenotypic resistance. This process can eventually result in more serious genetic resistance. Additionally, the bacterium encodes β‐lactamase enzymes that can inhibit β‐lactam antibiotics, contributing to drug resistance.

Acquired resistance:

During treatment of tuberculosis, chromosomal mutations in drug target genes can confer acquired resistance in *Mycobacterium tuberculosis*. These resistant strains are capable of transmission to others. The emergence of resistant mutants is influenced by several factors, including prolonged treatment duration, inconsistent adherence to prescribed regimens, medication shortages, and socioeconomic challenges in low‐income communities. These conditions can foster resistance to commonly used drugs and over time, lead to sequential resistance to additional therapies. Additionally, ineffective drugs or low doses of anti‐TB drugs during treatment can give the bacteria a survival advantage, selecting for resistant strains that multiply. This process can lead to mutagenesis and further increase drug resistance, ultimately resulting in multidrug‐resistant TB [17].

### 2.1 Isoniazid Resistance

Isoniazid (INH), formally known as isonicotinic acid hydrazide, features a pyridine ring and a hydrazine group [18]. INH and its derivatives have attracted attention in medicine for a range of biological activities, including antibacterial, antimycobacterial, and antiviral effects [19]. INH exhibits bactericidal properties specifically against *mycobacteria*, particularly during the rapid growth phase. However, it exerts a bacteriostatic effect in the latent or slow growth phase of bacteria [18]. INH serves as an effective prodrug in the first line of active tuberculosis (TB) treatment, which is activated by the catalase peroxidase *KatG* of *Mycobacterium tuberculosis* (*M. tuberculosis*) [20]. Upon activation, INH disrupts multiple essential processes in *M. tuberculosis*, notably interfering with lipid metabolism, protein synthesis, and nucleic acid synthesis. A key target is the synthesis of mycolic acids, which impairs cell wall formation and contributes to the bactericidal effect [21, 22].

Currently, INH is utilized as an antituberculosis drug. However, it is not used as a standalone treatment due to long‐term use, incorrect usage, and the presence of other point mutations. Nonetheless, it can be prescribed alone for prophylaxis [21]. Moreover, studies have indicated that INH can be effective in preventing stress‐induced necrosis, as well as halting the death and necrosis of infected neutrophils, thereby inhibiting active TB [23].

Initially, isoniazid (INH) was primarily used for the treatment of tuberculosis. However, resistance to *Mycobacterium tuberculosis* soon emerged [24]. Numerous studies have examined the resistance mechanisms of *M. tuberculosis* to INH, including mutations in *KatG*, *inhA*, *KasA*, *ndh*, and oxyR‐ahpC. Among these mutations, *KatG* appears to be the most influential in conferring INH resistance. In particular, the S315T mutation in the *KatG* gene has been identified as the most common mutation responsible for INH resistance in 90% of cases [21, 24].

This genetic mutation leads to a diminished capacity of *KatG* to catalyze the conversion of INH into isonicotinic acid, a crucial precursor required for the synthesis of the INH‐NAD complex [25].

Furthermore, it has been demonstrated that mutations leading to the overexpression of *inhA*, which encodes the enol‐acyl carrier protein reductase, also contribute to INH resistance [6]. Notably, the *inhA* gene is responsible for mycolic acid biosynthesis, which is hindered by INH through interference with the binding site in the *inhA* complex [18]. In fact, the proper functioning of INH relies on the formation of a covalent bond with NAD within the *inhA* binding site. Alterations to the NAD binding site and conformation can impact this process, leading to INH dependence [3]. Additionally, evidence suggests a direct correlation between certain transcription factors of the *TetR* family, particularly *rv0275* (*InbR*), and INH resistance. *Rv0275* has the ability to directly affect INH, and its overexpression is associated with resistance to the drug [15].

Similarly, overexpression of the *ahpC* gene, which is responsible for the alkyl hydroperoxide reductase enzyme in *M. tuberculosis*, also leads to INH resistance [14].

Unfortunately, the number of INH‐resistant *M. tuberculosis* strains is currently on the rise due to the prolonged and widespread use of the drug, as well as incomplete treatment courses [19]. Furthermore, it has been observed that increasing intracellular levels of inorganic polyphosphate strengthen resistance to INH [22]. Presently, one approach to mitigate INH resistance is the use of INH derivatives or combining INH with other drugs in treatment regimens. Additionally, prescribing INH alongside chemical compounds that interfere with the mechanisms of INH resistance can be effective [19, 22]. This issue carries significant importance due to the rapid increase in multidrug‐resistant tuberculosis (MDR‐TB) and extensively drug‐resistant tuberculosis (XDR‐TB), which is a cause for concern. Extensive studies and clinical trials should be designed to investigate various dimensions and strategies aimed at preventing the development and spread of INH resistance, while also enhancing the efficacy of INH in treating MDR‐TB and XDR‐TB across diverse populations and age groups.

The mechanism of resistance to isoniazid in Iran is mainly due to the following mechanisms, which include:

– Mutations in the *KatG* gene, especially at codons 315 and 335, result in reduced activation of isoniazid.
– Mutations in the *inhA* promoter region result in the overexpression of *InhA*, decreasing the potency of the drug4.
– Mutations in the *ahpC* gene serve a compensatory role against oxidative stress.
– Mutation in *KasA* gene (G269S) alters mycolic acid synthesis leading to resistance. (For further details, refer to Table 1).

**Table 1:** Common mutations of *Mycobacterium tuberculosis* in Iran.

Study area	Drug resistance status	Gene mutations	Reference
Iran (samples referred to the regional tuberculosis laboratory of Khuzestan province)	EMB	*embCAB*	[26]
		*embB306*	
		*embC270*	
Northwest of Iran (samples referred to the regional reference laboratory)	RIF	*RpoB*; Codon 531, 526, 516, 533, 522	[27]
Northern Iran, Golestan province	INH RIF	*RpoB*; Ser531Leu	[28]
		*KatG*; Ser315Thr	
		*InhA*; Promoter region	
Iran	RIF	*RpoB*; Ser531Leu	[29]
Iran, Tehran	INH RIF	*KatG*; Codon 315, 335	[30]
		*rpoB*; Codon 441	
West of Iran	EMB	*embB306*	[31]
		*embB406*	
Khuzestan province, Iran (specimens referred to the regional tuberculosis laboratory of Khuzestan province)	INH	*KatG*; Codon 315	[31]
		*InhA‐15*	
		*KasA*; G269S	
North east of Iran	Fluoroquinolone	*GyrB*; Codon 95	[32]
		*GyrA*; Codon 90, 91, 94	
Iran	EMB	*embB306*	[33]
		*embB366*	
Tehran	PZA	*pncA;* *A94V, E47G, P87L*	[34]
		*rspA;* *L105R, V97G, G104S*	
		*panD;* *D8G, A281V, L131R*	
		*inhA;* *promoter region*	
Isfahan	PZA	*pncA;* *E47G, P87L, L106P*	[34]
		*rspA;* *L105R, G104S, A140V*	
		*panD;* *D8G, L131R, A281V*	
		*inhA;* *promoter region*	
West of Iran	EMB	*embB;* *M306V, M306I, M306A, M306T, M306L*	[35]
		*embA*	
		*rpoB;* *S450L, H445N, L452P*	
		*KatG;* *S315T, S315N, S315I*	
		*inhA;* *promoter region*	
Tehran	INH	*KatG;* *S315T, S315N, S315I*	[36]
		*inhA;* *promoter region*	
		*ahpC*	
Isfahan	INH	*KatG;* *S315T, S315N, S315I*	[36]
		*inhA;* *promoter region*	
		*ahpC*	

Abbreviations: EMB; ethambutol; INH, isoniazid; PZA, pyrazinamide; RIF, rifampin.

### 2.2 Rifampin Resistance

Rifampin (RIF), also known as rifampicin, is a synthetic derivative of rifamycin that was introduced in 1972 as an antituberculosis medication [37]. The rifampin family includes RIF, rifalazil (KRM‐1648), rifabiotin (RBT), rifapentine (RPT), and CGP‐7040 [37]. RIF plays a crucial role in the treatment of tuberculosis due to its bactericidal properties [38]. It is considered one of the most effective first‐line drugs for the treatment of tuberculosis, specifically *Mycobacterium tuberculosis* [39, 40]. RIF works by targeting the DNA‐dependent RNA polymerase of the bacteria *Mycobacterium tuberculosis*, which is composed of four subunits α, β, β, and ϭ, encoded by the genes *rpoA*, *rpoB*, *rpoC*, *rpoD*, respectively [37]. By binding to the β subunit of the polymerase, RIF inhibits the initial phase of oligonucleotide chain elongation, leading to therapeutic effects. Since the RNA polymerase of *M. tuberculosis* is not similar to eukaryotic RNA polymerase, it is a suitable therapeutic target for treating *M. tuberculosis* [22].

Although resistance to RIF occurs less frequently compared to other drugs used in the treatment of tuberculosis, there has been an increase in the incidence of RIF resistance due to its widespread use [38]. Laboratory studies have reported that L2 strains of *M. tuberculosis* can acquire antibiotic resistance at a faster rate, and once they become resistant to INH, they are more likely to develop resistance to RIF [41].

The majority of resistance to RIF is caused by mutations in the *rpoB* gene. This gene encodes the β subunit of RNA polymerase in *M. tuberculosis*. More than 70 distinct mutations in the *rpoB* gene have been identified worldwide [42]. It is important to note that 96.7% of RIF‐resistant *M. tuberculosis* cases have been found to have a mutation in the *rpoB* gene [14].

*RpoB* plays a critical role in DNA transcription and mRNA synthesis. It utilizes four ribonucleoside triphosphates [25]. Several studies have shown that mutations in codons 516, 526, and 531 of the *rpoB* gene are the most commonly observed mutations in RIF‐resistant *M. tuberculosis* strains [25, 38, 40]. Among these mutations, the replacement of the amino acid serine with leucine at codon 531 is the most prevalent [25].

Furthermore, due to the structural similarities within the rifampicin family, mutations in codon 526 can lead to cross‐resistance between RIF and rifabutin [12].

In addition, a mutation in the *PonA1* gene has been identified, resulting in a twofold increase in the minimum inhibitory concentration (MIC) compared to the wild strain. Findings from previous studies suggest that this gene is involved in the synthesis of bifunctional penicillin‐binding protein and plays a role in cell wall synthesis. However, the specific mechanism of resistance to RIF remains unclear [43]. Laboratory conditions have also reported other mechanisms of resistance to RIF, in addition to chromosomal mutations, such as mistranslation of proteins resulting in phenotypic resistance to RIF. It has also been observed that *M. tuberculosis* resistant to RIF can transfer this drug from inside the cell using channels similar to calcium channels, which contributes to resistance [12, 13].

As previously mentioned, the prevalence of RIF‐resistant strains is rapidly increasing due to its extensive use as a first‐line treatment for tuberculosis. Statistics indicate that over 96% of these strains are attributed to mutations in the *rpoB* gene [44]. Further studies should be designed to more closely examine other causes of resistance to RIF and its mechanism on high‐scale population samples and on different races.

Mechanism of resistance of *M. tuberculosis* to the drug rifampin. Mutations of the *rpoB* gene that encodes the *β*‐subunit of RNA polymerase. The frequent mutations at codons 531, 526, 516, 533, and 522 cause conformational alterations, which enable rifampin to effectively bind to its target.

### 2.3 Resistance to Ethambutol

Ethambutol (EMB) was initially introduced in 1966 as a medication for *tuberculosis* and is currently utilized as one of the primary drugs in *tuberculosis* treatment [39]. It is also employed as a secondary treatment option for *tuberculosis* [45]. EMB, as one of the primary antituberculosis drugs, demonstrates effective bacteriostatic activity against *Mycobacterium* bacilli [46]. Structurally analogous to arabinose, EMB acts by inhibiting cell wall synthesis, thereby impeding the progression of *tuberculosis* [46, 47]. Arabinosyl transferase, involved in the biosynthesis of the cell wall components arabinosyl galactan and peptidoglycan, is another recognized target of EMB in cell wall synthesis [46, 48]. In fact, EMB enhances the permeability of the cell wall by hindering the formation of the mycolic acid‐arabinogalactan peptidoglycan complex, thereby facilitating the action of other antibiotics in tuberculosis treatment and playing a vital role in its efficacy [46]. It is recommended that EMB should not be used as monotherapy for tuberculosis treatment; instead, it should be administered in combination with other antituberculosis drugs to effectively combat *M. tuberculosis* and atypical *Mycobacteria*. EMB has also exhibited effectiveness against MDR strains of tuberculosis. Moreover, EMB is associated with lower toxicity compared to other primary drugs employed in *tuberculosis* treatment, particularly isoniazid [26]. Additionally, EMB is utilized as a secondary treatment option for tuberculosis [45].

Arabinosyl transferase is encoded by the *embCAB* locus, which comprises three genes: *embC*, *embB*, and *embA* [49]. Allelic studies have demonstrated that a mutation in the *embCAB* gene confers resistance to EMB in *M. tuberculosis* [47]. Previous research has indicated that the *embB* gene plays a role in arabinogalactan synthesis, while the *embC* gene is involved in lipoarabinomannan synthesis [49]. Numerous studies have established a significant correlation between EMB MIC and *embB* gene mutations [45]. The most frequently reported embB mutations include M306V, M306I, and G406A, with M306V being the most common [45]. Moreover, a mutation in codon 207 of the *embC* gene resulting in threonine‐isoleucine transfer has been observed in EMB‐resistant *M. tuberculosis* strains [49].

Another mechanism of EMB resistance involves *embR*. EmbR is essential for regulating *embCAB* transcription, and it's binding to the promoter region of the *embCAB* gene leads to increased expression and subsequent resistance to EMB [46, 50]. Furthermore, *embR* serves as a substrate for *pknH*, one of the serine‐threonine kinases in *M. tuberculosis*, which results in *embR* phosphorylation and upregulation of the *embCAB* operon [50]. Studies have also indicated that a mutation in *ubiA* (*Rv3806C*) causes high‐level resistance to EMB [47]. Specifically, mutations in codon 35 and 55 of *ubiA* lead to elevated dicaprylphosphoryl‐β‐d‐arabinose (DPA) levels, thereby competitively binding to the *embCAB* protein and resulting in EMB resistance [47]. Typically, EMB is prescribed in combination with RIF and INH to delay the emergence of resistance to both drugs [50].

EMB is one of the first‐line drugs in combination therapy against *M. tuberculosis*. Although the rapid increase in the occurrence of resistance among the strains of *M. tuberculosis* requires rapid detection of resistance to EMB [49], almost 50%–70% of tuberculosis resistant to EMB have mutations in codons 306–497 in the *embB* gene [51]. Resistance to EMB and mutations in its related genes are among the most important problems for patients because they lead to increased morbidity and mortality. Therefore, identifying the factors related to EMB resistance and determining the prevalence of EMB resistance in patients around the world is very important [52].

According to our extracted data from published papers from Iran, Ethambutol resistance is mainly due to mutations in the arabinosyl transferases genes, which are involved in the synthesis of the arabinogalactan layer in the bacterial cell wall:

– Codon 306, 366, and 406 mutations in the *embB* gene.
– Mutation of the *embC* gene at codon 270.
– Resistance can also arise through mutations in the *embCAB* gene cluster.

### 2.4 Resistance to Pyrazinamide

Pyrazinamide (PZA), which plays an important role in the treatment of tuberculosis, was discovered in 1952 [53]. PZA is a key drug in the current treatment regimen and future treatment regimens against M. tuberculosis. *In vivo*, this drug has the capacity to killing of the bacterial population but is not active against *M. tuberculosis* complex, which grow at neutral pH [54]. Pyrazine‐2‐carboxyamide (C5H5N3O) is a synthetic amide derived from pyrazinoic acid [55]. In fact, PZA is a structural analog of nicotinamide and a prodrug that is converted to the active form of pyrazinoic acid (POA) by the activity of pyrazinamidase (PZAase) enzyme [56]. POA is created as an anion in the cytoplasm, which has no antibiotic activity. Then POA is pumped out of the cell by the efflux pump and becomes protonated (HPOA) in the acidic environment. HPOA enters the cell and accumulates [57].

The accumulation of POA in the cell leads to the acidification of the cytoplasm and finally leads to the death of the bacteria. PZA is a crucial drug in the first‐line treatment of tuberculosis. It exhibits effective bactericidal activity against both antibiotic‐sensitive and MDR strains of tuberculosis [58]. PZA is used in combination with RIF and INH, resulting in a shortened treatment period from 9 to 6 months. Furthermore, PZA demonstrates effective activity against the latent form of *M. tuberculosis* [59]. Notably, PZA plays a vital role in the treatment of *M. tuberculosis* strains that are resistant to multiple drugs and is expected to remain a part of all treatment regimens in the foreseeable future [54].

Resistance to PZA typically arises due to mutations in various genes, including *pncA*, *rspA*, and *panD*, as well as other unidentified mechanisms [55]. Recent findings from the World Health Organization indicate that PZA resistance primarily results from mutations in the *pncA* gene and its promoter region [55]. Studies indicate that 72%–98% of PZA‐resistant strains are associated with a mutation in the *pncA* gene [58]. These mutations lead to a deficiency in the activity of the PZAase enzyme, allowing *M. tuberculosis* to survive in the presence of PZA [57]. Noteworthy mutations in this gene associated with resistance include Cys138, Asp68, and Gln141 in the active site of the enzyme [55]. In strains exhibiting low levels of PZA resistance while maintaining PZAase activity, it is presumed that other mechanisms of resistance to PZA are at play [56].

The findings of these studies suggest the possibility of other genomic regions outside the *pncA* gene being involved in PZA resistance. One such gene is *rspA*, which encodes ribosomal protein S1 [57]. This protein binds to the ribosome and facilitates the trans‐translation process by directing mRNA sequences upstream [55]. Mutations in *rspA* can alter the binding site of PZA and subsequently confer resistance [58]. Most mutations in PZA‐resistant species have been observed in codons 973–1051, specifically in codons 1024‐1030 of the *rspA* gene [55].

Additionally, mutations in the *panD* gene, which encodes aspartate α‐decarboxylase, have been reported in PZA‐resistant *M. tuberculosis* [56]. Aspartate α‐decarboxylase is responsible for the biosynthesis of β‐alanine, a precursor for pantothenate and coenzyme A [55]. A translocation mutation of Ser134 in the *panD* gene has been identified in multidrug‐resistant *M. tuberculosis* strains associated with PZA resistance [55]. Furthermore, overexpression of the panD gene has been linked to resistance against PZA and its active component, POA [56].

There is a suggestion that mutations in the *clpc1* gene may be related to the occurrence of resistance to POA. The *clpc1* gene encodes *clpc1*, an ATPase that functions as a chaperone for the essential cytoplasmic protease Clp. However, the precise relationship and mechanism by which this gene influences resistance to POA remain unclear [60]. Additionally, there is a proposal that POA inhibiting fas1 may play a role in the development of resistance to PZA [57].

Many efforts have been made to understand the molecular mechanism of resistance to PZA [54]. Increased resistance to PZA has been observed in *Mycobacterium tuberculosis* strains that resistant to multiple drugs, which can be attributed to previous treatment regimens received by patients and the development of cross‐resistance to PZA. In addition, the role and efficacy of PZA in patient treatment should be carefully considered [58]. Therefore, further studies with a large sample size and comprehensive design should be conducted to explore other mechanisms and gene mutations involved in PZA resistance.

Mutations in pyrazinamide‐conversion and metabolism‐related genes are associated with pyrazinamide resistance:

Mutations in the *pncA* gene (A94V, E47G, P87L, L106P) cause the chemical conversion of pyrazinamide not to generate its active metabolite pyrazinoic acid.

Mutations on the *rspA* gene (L105R, V97G, G104S, A140V) disrupt ribosomes.

Mutations in the *panD* gene (D8G, A281V, and L131R) affect coenzyme A biosynthesis that alters bacterial survival under the drug pressure.

### 2.5 Efflux Pumps and Antibiotic Resistance

Efflux pumps are membrane proteins capable of actively transporting various substances, including antibiotics, from the cytoplasm to the extracellular environment [61]. *Mycobacterium tuberculosis* employs diverse mechanisms to develop antibiotic resistance, with gene mutations being the most common [62]. Efflux pumps, which contribute significantly to antibiotic resistance in *M. tuberculosis* strains lacking genetic mutations, have been documented [63]. Recent evidence indicates that efflux genes are upregulated upon drug exposure and play a role in antibiotic resistance [64]. Increased efflux pump activity in response to drug exposure represents the initial step in the emergence of antibiotic resistance [61]. Efflux pump genes and their products have been investigated in *M. tuberculosis* regarding their presence and activity. Some of these pumps are more pronounced in drug‐resistant *M. tuberculosis* strains when exposed to the drug, while others show reduced expression in the absence of medication [62].

Efflux pumps in bacteria can be classified into five major families based on their homologous sequences, including the ATP‐binding cassette superfamily (ABC), the major facilitator superfamily (MFS), the multi‐drug and toxic compound extrusion family (MATE), the resistance‐nodulation‐cell division superfamily (RND), and the small multi‐drug resistance family (SMR). The genome of *M. tuberculosis* encodes a wide array of efflux pumps, predominantly belonging to the large ABC and MFS families, which contribute to antibiotic resistance [65]. Indeed, the expression of these efflux pumps in *M. tuberculosis* represents the initial step in the development of drug resistance, ultimately leading to chromosomal mutations associated with drug resistance [2].

Approximately 30% of isoniazid‐resistant strains and 3% of rifampicin‐resistant strains do not exhibit any known genetic causes, which may be attributed to the presence of efflux pumps. Efflux pumps have the ability to transport a variety of structurally distinct substances out of the cellular environment. Moreover, it has been duly demonstrated that efflux pumps play a vital role in the intracellular proliferation of *M. tuberculosis* within macrophages, effectively extruding nearly all antituberculosis medications from the cell [66]. Furthermore, the presence of medicinal efflux is intricately linked to pathogenicity, virulence, biofilm formation, and the modulation of specific mutations in the genes responsible for encoding *M. tuberculosis* efflux pumps or drug resistance. Additionally, it is conceivable that these genes can undergo excessive expression as an acquired mechanism in drug‐sensitive strains of *M. tuberculosis* in response to drug exposure. The expression of the efflux pump is contingent upon environmental conditions and is reversible and temporary [67]. Research has demonstrated that long exposure of *M. tuberculosis* to INH can damage efflux systems, resulting in an increased resistance phenotype. It has also been reported that the *iniA* gene is necessary for efflux pump activity, which leads to resistance to both INH and EMB. Furthermore, overexpression of large ABC family transporters, including Rv1819c and *pstB* [*Rv0933*], has been observed in relation to INH resistance [68]. Guilian Li et al. found differential gene expression of *drrA*, *drrB*, *mmr*, *efpA*, *jefA* (*Rv2959*), *Rv0849*, *Rv1634*, and *Rv1250* induced by INH/RIF in *M. tuberculosis*. *DrrA* has been proposed as an important factor in INH resistance, as well as for low‐level resistance to RIF. Additionally, the expression of *jefA* (*Rv2959*) exposed to INH is 4 to 4.5 times higher than normal [62]. Increased expression of *jefA* (*Rv2959*) may contribute to elevated resistance to EMB and INH in *M. tuberculosis* [69]. In XDR strains, Narang et al. found a mutation at position 1,839,306 in *Rv1634*, which is dominant for *rpsl*, *rrs*, *gidB*, or *drrA* [68]. Laws et al. have also indicated that several other efflux pumps from the large ABC family, such as *Rv0194*, *Rv1819c* (*BacA*), and *Rv2936*/*Rv2937*/*Rv2938* (*DrrABC*), play a role in MDR strains of *M. tuberculosis*. These pumps transport various substances, including chloramphenicol, macrolides, and tetracycline, out of the cell [65]. *Mmr* (*Rv3065*) is an efflux pump from the SMR family, and its gene expression significantly increases when exposed to INH. To date, *Mmr* is the only member of the SMR family that has been reported to be involved in drug resistance in *M. tuberculosis* [61, 65]. The efflux protein *Rv1258c*, also known as Tap or P55, is overexpressed upon exposure to INH and RIF. This overexpression is believed to contribute to drug resistance in *M. tuberculosis*. Point mutations V219A and S292L in the efflux pump Tap (*Rv1258c*) have been shown to cause drug resistance in *M. tuberculosis* against several drugs, including INH and PZA [64]. As mentioned, efflux pumps are located in the membrane of *M. tuberculosis* [62]. Ever since the initial discovery of the involvement of efflux pumps in drug resistance, these mechanisms have been recognized as major contributors to bacterial drug resistance, ultimately leading to the emergence of MDR strains. The antibiotic resistance of *M. tuberculosis* due to INH exemplifies the claim that resistance can arise from gene mutations or mechanisms related to the efflux pump [68]. Exposure of *M. tuberculosis* to sublethal INH concentrations can upregulate efflux pump activity, facilitating drug expulsion and enabling survival [62]. Recent investigations into the role of efflux pumps in *M. tuberculosis* antibiotic resistance have proposed efflux pump inhibitors as a therapeutic strategy [63]. However, while such inhibitors may mitigate resistancedevelopment, they could also diminish antibiotic efficacy [61]. Further research is needed to achieve a more comprehensive understanding of efflux pump mechanisms and their contribution to tuberculosisresistance. Additionally, large‐scale studies should include diverse populations across age, gender, and race to inform treatment strategies.

## 3 New Therapeutic Approaches

### 3.1 Herbal Treatment

In recent years, attention and interest in medicine based on natural products have increased [70]. Plants, which contain a variety of biologically active compounds, play an important role in the treatment of many human diseases. Seventy to ninety‐five percent of the world's population use traditional medicine to meet their basic medical needs [71]. The urgent need for new developments to reduce the burden of tuberculosis has been well demonstrated in recent studies. Additionally, new anti‐mycobacterial drugs based on herbal products have also been investigated in studies [72]. The discovery of new drugs basically includes the identification of new chemical capacities that have the characteristics required for drug resistance and medicinal chemistry [73].

Plants are one of the sources of biologically active secondary metabolites that have high therapeutic potential [70]. Some of these secondary metabolites, including *alkaloids*, *coumarins*, *flavonoids*, and *polyphenols*, are widely used due to their activity against a wide range of human diseases [71].

Selecting plants for their biological activity is the first and most important step in the drug discovery program [70]. This is very important in line with the WHO goal, which is based on a 95% reduction in deaths due to tuberculosis and a 90% reduction in tuberculosis by 2035. Plants have also been considered in the development of antituberculosis drugs due to their high potential in the development of plant‐based drugs [72].

Many studies on the effect of medicinal plant extracts against *M. tuberculosis* H37Rv have been reported in laboratory conditions. The plant *Lantana hispida kunth*, which is traditionally used in the treatment of respiratory diseases, is effective against some MDR strains [70]. It has been shown that chemical substances derived from plants have a better effect in preventing the growth of *Mycobacterium* and have fewer side effects, and it is possible to ensure faster recovery of patients [71]. The active plant compounds of neir can be used to increase the efficiency and effectiveness of existing drugs [73]. Additionally, it has been shown that a combination of plant extracts and RIF has a better and more efficient effect against RIF/streptomycin resistant strains [71].

*Plumericin phytomolecules* purified from *Plumeria bicolor* plant have activity against sensitive strains as well as MDR strains of tuberculosis. The active compounds have shown better performance than INH and RIF against MDR strains of *tuberculosis*. The active compound ursolic acid, which is prepared from the medicinal plants *Chamaedoria tepejilote liebm* and *Lanta hispida kunth*, has antituberculosis activity against *M. tuberculosis* infections in macrophages [70]. Curcumin, with its effect on human macrophages, probably through the positive regulation of apoptosis and autophagy through the activation of caspase 3, strengthens their activity against *M. tuberculosis* [74].

### 3.2 Nanomedicine

Which is one of the most important components of nanotechnology, has created a huge revolution in facilitating targeted drug delivery in infectious diseases such as tuberculosis and global health [75]. Nano medicine is the engineering and designing colloidal particles with less than one‐micron size. To generate this particle, natural, semi‐synthetic, and synthetic polymers are utilized. Drugs are as enmesh or solid solutions in a polymer matrix trapped or encapsulated [76].

Nanoparticles have innated antimicrobial proprieties, which results in a reduction of dosing regimen and significant decreased associated side effects; in addition, nanoparticles reduce size, increase the surface‐to‐volume ratio and provide the possibility to change the therapeutic protentional of drugs [77]. Drug carriers based on nanomedicine exhibit high stability, significant payload capacity, and the ability to load both therapeutic factors which means hydrophilic and hydrophobic. Additionally, they enable various administration routes, such as oral and inhalation. Also, they can provide controlled and prolonged released therapeutic factors, particularly polymeric and lipidic nanoparticles [75]. Collectively, the unique physiochemical properties of nanomedicine, such as multifunctionality, nanoscale size, and high reactivity, have made it a preferred choice for clinical use [75]. In nanomedicine, drugs are encapsulated within a polymer matrix or attached to a nanoparticle surface through absorption or chemical interactions. Nanoparticles can target specific sites in the drug delivery system and enable controlled drug delivery. This technology improves the efficiency of drugs by enhancing their stability in blood circulation and their biodistribution [76].

Nanoparticles can selectively deliver high concentrations of drugs to macrophages, the primary host cells, thereby preventing potential drug side effects in *tuberculosis* transmission [78]. These nanoparticles act as protective carriers, shielding drugs from degradation and facilitating targeted drug delivery to macrophages. Additionally, the use of nanoparticles reduces toxicity and helps prevent antibiotic resistance mechanisms [79].

Studies have shown that nanoparticles are more effective than free drugs in treating *tuberculosis* [78]. In the 1990s, nanotechnology was identified as a promising solution for tuberculosis treatment [79]. Nanoparticles connect to the determined specific site and regulate drug delivery. TB granuloma including *M. tuberculosis* infected macrophages is surrounded by immune cells, fatty acids, and cholesterol. In this granuloma vascularized is powerless which means delivering drugs into the granuloma is challenging. Nanoparticles, owing to their small size, similar to intracellular molecules like DNA and ease of phagocytosis by macrophages, have the capacity to target intracellular *M. tuberculosis* [76].

In recent years, drug delivery systems capable of delivering antituberculosis drugs to specific controlled sites have emerged. The Poly lactic‐co‐glycolic acid (PLGA) nanoparticle drug delivery system has garnered significant attention recently. PLGA has been proposed as one of the most advanced biodegradable polymers, demonstrates adjustable degradation periods based on its molecular weight and copolymer composition. Its solubility in common solvents allows it to be molded into various forms. This versatility has led to its extensive testing as a carrier for drugs, proteins, and macromolecules like DNA, RNA, and peptides. Incorporating ethionamide into PLGA matrices extends drug‐release durations in tuberculosis therapy, potentially improving patient adherence to treatment regimens. Conversely, moxifloxacin (MOX), a DNA gyrase inhibitor, can contribute to rapid clearance of *M. tuberculosis*; however, sustained efficacy necessitates ongoing administration, which may be associated with adverse effects such as hepatotoxicity and the emergence of resistance. To address these limitations, PLGA‐based nanoparticles offer a promising platform for controlled and efficient delivery of MOX in tuberculosis treatment. Clofazimine (CFZ) is highly effective against MDR *tuberculosis* strains, but due to low drug bioavailability after oral administration, it exhibits reduced therapeutic efficacy. To overcome this challenge, PLGA‐Polyethylene Glycol nanoparticles loaded with CFZ have been recommended for treating central nervous system tuberculosis, resulting in a significant reduction in drug toxicity [80].

INH is a first‐line drug for the treatment of tuberculosis and works against both active and latent *tuberculosis*. Side effects include neurotoxicity and severe liver damage, which are caused by the drug's effect on nerve cells and hepatocytes instead of macrophages. Also, this leads to a decrease in the effective concentration of antibiotics at the location of macrophages, which in turn leads to antibiotic resistance. Therefore, a nanoparticle‐based platform for tuberculosis drugs has the capacity to protect antibiotics from hepatic and renal clearance and deliver them correctly to the site of infection, acting more effectively than the free drug and preventing also reduce antibiotic resistance. In fact, the protection antibiotics through nanoparticles, saves them from degradation by immune system results in prolonged drug release, delayed drug clearance, increased chance of reaching infection site and favorable pharmacokinetics [78]. On the other hand, by connecting nanotubes to drugs such as INH by chemical reactions, more efficiency of these drugs can be seen, because these drugs cannot penetrate the cell wall of *Mycobacterium* and with the help of these nanotubes, these limitations can also be overcome [81]. It has also been reported that the use of multi‐walled carbon nanotubes (MWCNTs) leads to a reduction in the dose of INH required for treatment and a reduction in the incidence of drug resistance [82] (for more information, see the Table 2).

**Table 2:** Advantages and disadvantages of nanomedicines.

Study	Date	Disadvantages	Advantages	Nano drug types discussed
Borah slater et al. [76]	2023	Challenges in production and processing Cost Toxicity	High physicochemical facilities Multifunctional nano scale Improved drug delivery Improved patient compliance	Polymeric Nano drug Liposomes
Hatae et al. [79]	2023	Lack of information on long‐term side effects Potential drug leakage Stability issue Cost	Selective drug delivery Increasing drug efficiency Biocompatibility Biodegradability	Liposomes Solid lipid nanoparticles (SLNs) Nanostructured lipid carriers (NLCs)
Shao et al. [80]	2023	Problems in large‐scale production and cost and toxicity Limited clinical application	Controlled drug release Increased bioavailability Reduced drug resistance	Nanocarriers based on PLGA
Sheikhpour et al. [81]	2022	Concerns about biodistribution and long‐term safety Toxicity Drug release Cost	Increasing drug efficiency Selective drug delivery Reduced drug toxicity	Carbon nanotubes Liposomes
Chaudhary et al. [75]	2022	Report lack of specificity Cost Stability	Facilitate controlled drug delivery to the site of infection Enhanced drug solubility Controlled drug release Reduced drug toxicity	Polymeric nanoparticles Lipid nano particles Metallic nano particles Phyto fabricated nanoparticles Ligand and anchored nanoparticles Nonembedded microparticles Multi‐functionalized nanoparticles
Zomorodbakhsh et al. [82]	2022	Concerns about drug toxicity and safety Cost Stability	Reduced drug dose Potential to overcome drug resistance Reduced drug toxicity Enhanced drug delivery	Multi‐walled carbon nanotubes (MWCNT)
Kerry et al. [83]	2017	Ethical considerations Toxicity Cost Stability	Different applications Opportunities for medical innovation Enhanced drug delivery and efficacy Reduced drug toxicity	Liposomes Dendrimers Nanotubes
Hwang et al. [78]	2015	Possible toxicity Challenges in clearance from the body Drug releases Cost	Improved drug delivery Drug absorption protection Controlled release	Carriers of antituberculosis nanomedicines: pH ‐responsive INH‐ loaded nanoparticles

The current regimens for the treatment of tuberculosis are long and include 6–9 months of treatment with several drugs and are very complicated due to the occurrence of drug resistance and drug toxicity. Nanoparticle drug delivery systems, which directly carry very high concentrations of antibiotics to the site of infection, promise to improve treatment by eliminating the causative agent faster and better [78]. The effectiveness of nanomedicine in the performance of drug‐sensitive tuberculosis strains has been successfully demonstrated by various studies [76]. In fact, recent advances in technology, especially nanotechnology, have provided new opportunities for medical science to treat diseases with the synthesis of nanoparticles [83]. Further studies should be conducted to investigate the efficiency of these nanoparticles against MDR and XDR strains. Tuberculosis should be designed on different populations and races.

## 4 Conclusion

*M. Tuberculosis* has assumed alarming proportions with the increasing spread of antibiotic‐resistant cases. The current status of antibiotic resistance of *M. Tuberculosis* is a serious threat to global health with the emergence of MDR and XDR strains every day.

Knowledge of resistance mechanisms in *M. tuberculosis* is not only crucial for the global community but also a significant step in the development of new drugs and treatment strategies against tuberculosis. Resistance to antibiotics in this bacterium has become a serious challenge, and there is a need to implement immediate control measures to prevent the spread of resistant strains of *M. tuberculosis*.

As discussed earlier, the emergence of INH resistance, primarily through genetic mutations in genes like *KatG* and *inhA*, poses a significant challenge. In Iran, resistance mechanisms include mutations in the *KatG* gene, *inhA* promoter region, *ahpC* gene, and *KasA* gene. These mutations reduce INH activation and potency. To combat resistance, strategies include using INH derivatives, combination therapies, and compounds that interfere with resistance mechanisms. Extensive research and clinical trials are needed to prevent the spread of INH resistance and enhance treatment efficacy. Furthermore, RIF is essential in TB treatment due to its bactericidal properties. However, increasing resistance, mainly due to *rpoB* gene mutations, presents a significant challenge. Common mutations at codons 516, 526, and 531 hinder RIF's effectiveness. The rise in RIF‐resistant strains is linked to widespread use and cross‐resistance with other rifamycins. In Iran, similar resistance mechanisms involving *rpoB* gene mutations are observed, compounding the treatment challenge. Addressing RIF resistance requires further research, rapid detection methods, and new treatment strategies to improve TB control globally and in regions like Iran. The other anti‐TB drug, EMB is vital in treating TB due to its bacteriostatic activity and low toxicity However, resistance to EMB is rising, driven by mutations in the *embCAB* gene locus, especially in *embB* and *embC* genes. In Iran, EMB resistance is primarily due to mutations in the arabinosyl transferase genes, affecting the arabinogalactan layer synthesis in the bacterial cell wall. Key mutations occur at codons 306, 366, and 406 in *embB* and codon 270 in *embC*. To combat EMB resistance, rapid detection methods and a deeper understanding of genetic factors are crucial. Identifying the prevalence and factors of EMB resistance is essential to improving treatment outcomes and managing drug‐resistant TB globally and in regions like Iran. PZA is crucial in TB treatment due to its bactericidal activity against both drug‐susceptible and multidrug‐resistant (MDR) strains. PZA is activated to pyrazinoic acid (POA), which disrupts bacterial metabolism and leads to cell death. However, resistance to PZA is a growing concern, primarily driven by mutations in the *pncA* gene, with additional contributions from *rspA* and *panD*. In Iran, PZA resistance is largely attributed to *pncA* mutations that impair conversion of PZA to its active metabolite POA, with further involvement of *rspA* and *panD* affecting ribosomal function and coenzyme A biosynthesis. Addressing PZA resistance necessitates further research to identify additional resistance mechanisms and associated gene mutations. Understanding these factors is essential for developing effective treatment strategies and mitigating drug‐resistant TB globally and in regions such as Iran.

Based on the findings of the present study, there is an urgent need for the development and evolution of novel therapeutic agents against *M. tuberculosis*. Ensuring completion of drug regimens and implementing new strategies to prevent resistance are critical for addressing MDR and XDR strains. Accelerating the process of developing new drugs, effective compounds, and innovative approaches in the treatment of tuberculosis can be an effective step in preventing the spread of resistance and enhancing treatment efficiency. Considering the necessity to develop new solutions and drugs to combat the spread of antibiotic resistance in tuberculosis, further studies should be conducted on a large‐scale population sample across different races with diverse geographical distributions.

## Author Contributions

**Ali Bayat Bodaghi:** conceptualization, data curation, formal analysis, visualization, writing – original draft, methodology, investigation, supervision, validation, funding acquisition, writing – review and editing. **Aref Shariati:** data curation, conceptualization, investigation, supervision, validation, visualization. **Jebreil Shamseddin:** writing – review and editing, writing – original draft, project administration, data curation, investigation, validation, visualization. **Amir Bayat Bodaghi:** investigation, data curation, writing – original draft, validation. **Mina Rezaei:** writing – review and editing, investigation, validation. **Abbas Farahani:** conceptualization, data curation, formal analysis, visualization, writing – review and editing, project administration, supervision, investigation, validation, funding acquisition.

## Conflicts of Interest

The authors declare no conflicts of interest.

## Transparency Statement

The lead author Abbas Farahani affirms that this manuscript is an honest, accurate, and transparent account of the study being reported; that no important aspects of the study have been omitted; and that any discrepancies from the study as planned (and, if relevant, registered) have been explained.

## Data Availability

All data generated and analyzed during the current study are available from the corresponding author on reasonable request.
